# Structure of the Parainfluenza Virus 5 (PIV5) Hemagglutinin-Neuraminidase (HN) Ectodomain

**DOI:** 10.1371/journal.ppat.1003534

**Published:** 2013-08-08

**Authors:** Brett D. Welch, Ping Yuan, Sayantan Bose, Christopher A. Kors, Robert A. Lamb, Theodore S. Jardetzky

**Affiliations:** 1 Department of Molecular Biosciences, Northwestern University, Evanston, Illinois, United States of America; 2 Howard Hughes Medical Institute, Northwestern University, Evanston, Illinois, United States of America; 3 Department of Structural Biology, Stanford University School of Medicine, Stanford, California, United States of America; Institut Pasteur, France

## Abstract

Paramyxoviruses cause a wide variety of human and animal diseases. They infect host cells using the coordinated action of two surface glycoproteins, the receptor binding protein (HN, H, or G) and the fusion protein (F). HN binds sialic acid on host cells (hemagglutinin activity) and hydrolyzes these receptors during viral egress (neuraminidase activity, NA). Additionally, receptor binding is thought to induce a conformational change in HN that subsequently triggers major refolding in homotypic F, resulting in fusion of virus and target cell membranes. HN is an oligomeric type II transmembrane protein with a short cytoplasmic domain and a large ectodomain comprising a long helical stalk and large globular head domain containing the enzymatic functions (NA domain). Extensive biochemical characterization has revealed that HN-stalk residues determine F specificity and activation. However, the F/HN interaction and the mechanisms whereby receptor binding regulates F activation are poorly defined. Recently, a structure of Newcastle disease virus (NDV) HN ectodomain revealed the heads (NA domains) in a “4-heads-down” conformation whereby two of the heads form a symmetrical interaction with two sides of the stalk. The interface includes stalk residues implicated in triggering F, and the heads sterically shield these residues from interaction with F (at least on two sides). Here we report the x-ray crystal structure of parainfluenza virus 5 (PIV5) HN ectodomain in a “2-heads-up/2-heads-down” conformation where two heads (covalent dimers) are in the “down position,” forming a similar interface as observed in the NDV HN ectodomain structure, and two heads are in an “up position.” The structure supports a model in which the heads of HN transition from down to up upon receptor binding thereby releasing steric constraints and facilitating the interaction between critical HN-stalk residues and F.

## Author Summary

Paramyxoviruses comprise a large family of significant pathogens including Newcastle disease virus (NDV), parainfluenza viruses 1-5 (PIV1-5), respiratory syncytial virus, the highly transmissible measles virus, and the emerging and deadly Nipah and Hendra viruses. Five paramyxoviruses are U.S. Department of Health and Human Services and U.S. Department of Agriculture “select agents,” and prevention and/or treatment of these viruses is a public health priority. Paramyxoviruses infect host cells through the concerted action of a “mushroom-shaped” receptor binding protein (HN, H, or G) and fusion protein (F) on the viral surface. However, despite numerous biochemical and structural insights, many details remain unknown about how these proteins interact and the mechanism by which the interaction triggers membrane fusion. Here we present the X-ray crystal structure of the PIV5 HN ectodomain comprised of a large fragment of the stalk and complete head domains. The structure reveals a unique conformation that is a hybrid of that seen in previous NDV ectodomain and PIV5 attachment protein head domain structures. A high-resolution view of the different orientations that head domains can adopt combined with recent biochemical data suggest a simple mechanism for paramyxovirus fusion. These new insights will help guide vaccine and inhibitor discovery efforts for paramyxoviruses.

## Introduction

The *Paramyxoviridae* are membrane-enveloped negative-sense single-stranded RNA viruses that infect animals and humans often resulting in significant disease and mortality. Most paramyxoviruses enter cells at neutral pH by fusing their envelope with the plasma membrane of a target cell thereby releasing a ribonucleoprotein complex into the cytoplasm. Paramyxovirus fusion is typically mediated by two glycoproteins on the surface of virions: a trimeric fusion protein, F, with type I viral fusion protein characteristics, and a receptor binding protein variously named HN, H, or G depending on the virus and protein functionality [1].

Viruses with hemagglutinin-neuraminidase (HN) attachment proteins use sialic acid as a receptor and include parainfluenza virus 5 (PIV5), Newcastle disease virus (NDV), mumps virus, human parainfluenza viruses (hPIV1-4), and Sendai virus. HN proteins have at least three functions: (1) they bind sialic acid receptors on glycoproteins and gangliosides at the cell surface (hemagglutinin activity). This function is thought to play an important role in timing and initiating virus-cell fusion. (2) HN proteins are neuraminidases, which catalyze the hydrolysis of glycosidic linkages on terminal sialic acid residues thus destroying the receptor. Neuraminidase activity likely plays a crucial role removing sialic acid from viral and cellular conjugates during assembly and budding [2]. (3) A function common to paramyxovirus attachment proteins is to lower the activation barrier of the F protein, presumably through direct interaction, thereby triggering a major refolding event in F from a metastable prefusion form [3], [4], [5] to a highly stable post-fusion form [6], [7], [8]. The merging of viral and target cell membranes is coupled with this structural rearrangement [9].

PIV5 HN is comprised of a short cytoplasmic tail (residues 1–17) at its N-terminus, a transmembrane domain (residues 18–36), and a large ectodomain (residues 37–565). The ectodomain is composed of a helical stalk and a large globular head containing the hemagglutinin/neuraminidase (NA) active site(s). The X-ray crystal structures of the globular head domain of NDV, hPIV3, PIV5, measles virus (MeV), Hendra virus (HeV), and Nipah virus (NiV) reveal a six-bladed beta-propeller fold typical among neuraminidases [10], [11], [12], [13], [14], [15], [16], [17]. For PIV5, a sialyllactose receptor-bound globular head domain structure revealed that sialyllactose binds in the center of the beta propeller [17].

These structures, as well as biochemical data [18], reveal that PIV5 HN forms covalent dimers (via Cys 111) that further assemble into a non-covalent dimer of dimers via the stalk domain [19] with possible contribution from the cytoplasmic tail and transmembrane domains [20]. Recent crystal structures of both the NDV-HN ectodomain (including stalk residues 79–115 and head domains) and the isolated stalk domain of PIV5 HN (residues 56–108) reveal a four-helix bundle (4HB) stalk with robust hydrophobic core packing [16] [21]. Additional studies have shown that attachment proteins of other paramyxoviruses have stalks consistent with a 4HB structure [22], [23], [24],[25].

A substantial body of evidence including point mutations, additions of glycan moieties, chimeras, insertions, and truncations has established that residues in the stalk affect fusion promotion [21], [24], [26], [27], [28], [29], [30], [31], [32], [33], [34], [35], [36]. For NDV HN and several paramyxoviruses that utilize protein receptors, stalk mutations that block fusion have also been shown to disrupt coimmunoprecipitation with F [34], [27], [37], [36].

**Figure 1:**
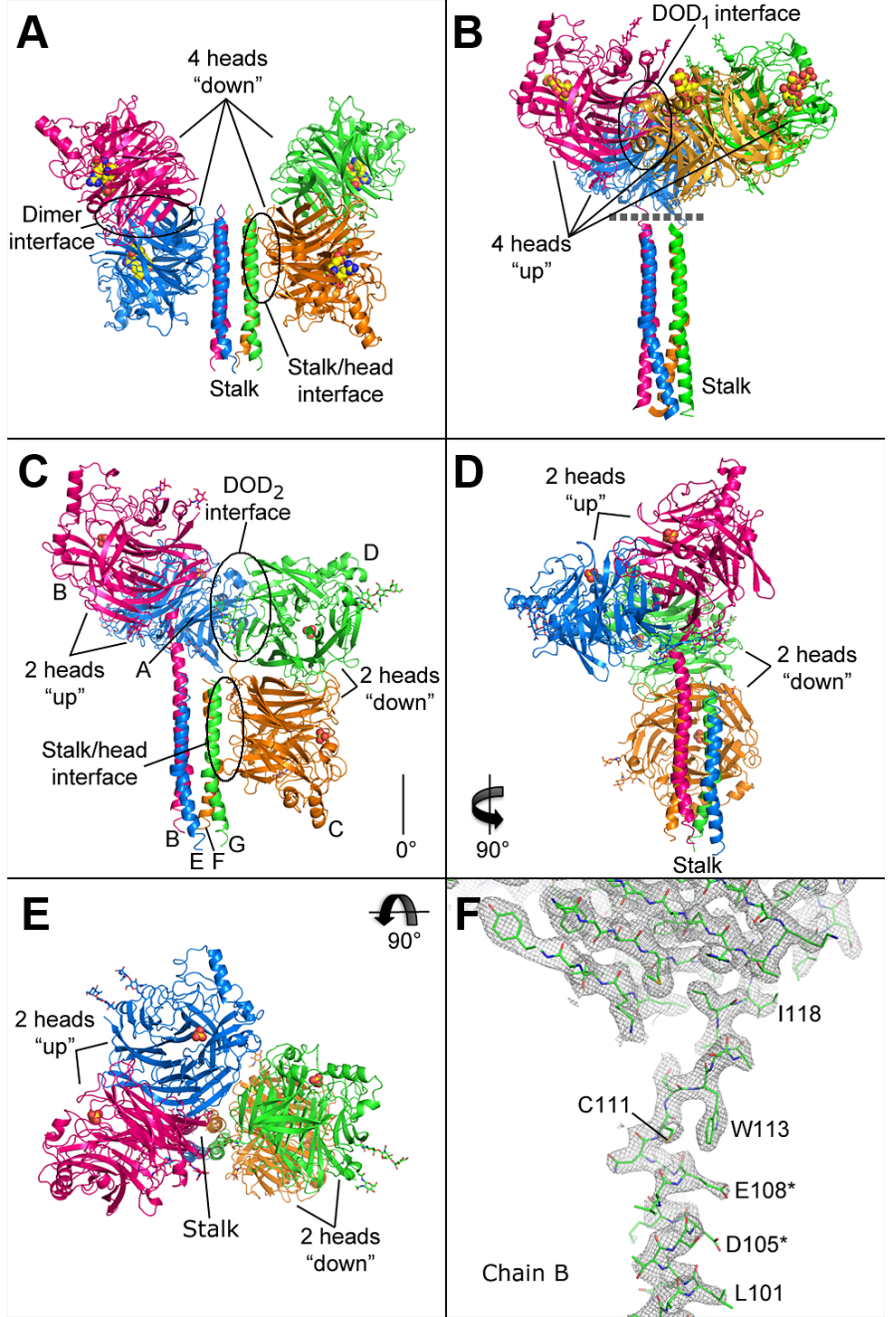
Crystal structures of various HN ectodomain conformations. A) Cartoon representation of the NDV HN crystal structure (PDB ID: 3TIE) in the “4-heads-down” conformation. Active site residues (E400, R415, and Y525) are shown as yellow, blue and red spheres. The locations of the second sialic acid binding sites are not shown. B) Model of the “4-heads-up” conformation of PIV5 HN comprised of separate previously solved crystal structures of the head domain (PDB ID: 1Z4X) and the 4HB stalk (PDB ID: 3TSI). Sialyllactose in the active site is shown as yellow and red spheres. Ordered glycan chains are shown as sticks. Coloring as in A. C–E) Front, side, and top views, respectively, of the “2-heads-up/2-heads-down” conformation revealed by the PIV5-HN ectodomain crystal structure. A sulfate ion coordinated by R405, R495, and Y523 in each active site, which partially overlaps with sialyllactose in the head domain of the 1Z4X structure, is shown as orange and red spheres. Ordered glycan chains are shown as sticks. Coloring as in A–B. F) Sigma-A weighted 2Fo-Fc electron density map rendered at 1.0 sigma showing the fit of the final PIV5-HN ectodomain model. D105 and E108, project charged sidechains toward the central 4HB axis and are marked with an asterisk. The region shown is the continuous density that links the stalk of chain B to its globular head domain. The PIV5-HN ectodomain structure was used to position head domains relative to the stalk in the 4-heads-up model in B.

The crystal structure of the NDV HN ectodomain [16] revealed an interface between the HN heads and the upper portion of one side of the stalk 4HB, in a conformation we refer to as “4-heads-down” (Fig. 1A). Interestingly, the NDV heads largely obscure the region of the stalk implicated by mutagenesis as forming direct interactions with F on two sides of the 4HB. The PIV5 HN stalk structure [21] revealed a similar 4HB and potential site for F interactions, but since that structure did not include the HN head domains, evidence for PIV5 HN head-stalk interactions has been lacking. We recently showed that the head domains of PIV5 HN are completely dispensable for fusion activation. A construct lacking the head domains (“headless HN”) activates fusion at levels comparable to wild-type (wt) HN [38]. Together, these results suggested a model for fusion activation in which the ‘4 heads down’ HN represents a prefusion conformation that restricts or blocks F interactions with the stalk and that deletion of the head domains allows F interactions that promote membrane fusion [38].

The NDV ectodomain structure also revealed a novel organization of the four head domains relative to one another as compared to those previously observed in crystal structures of other paramyxovirus attachment protein head domains [16]. Regardless of the absence or presence of the stalk domain in the expression constructs used to solve NDV-, hPIV3-, and PIV5-HN head domain structures, the head domains for each of these viruses formed a similar dimeric arrangement, except for a single low pH form of the NDV HN protein [12]. A dimer-of-dimers (DOD) tetramer was observed in a previous PIV5 HN ectodomain crystal structure, and a similar arrangement was observed in NDV neuraminidase domain crystals (Figs. 1B, S1) [12], [14], [17]. Unlike, the NDV HN 4-heads-down conformation, the original PIV5 tetramer arrangement places N-termini relatively close together on one side of the tetramer such that a stalk could extend down from the center toward the viral membrane. This tetrameric arrangement could represent a post-receptor binding state for HN, which we refer to as the “4-heads-up” conformation. A composite model of this hypothetical conformation can be generated using the separately determined PIV5 HN head domain and 4HB stalk structures as shown in Fig. 1B.

NDV HN heads can adopt different arrangements in different crystal forms, and electron microscopy data indicates that the heads of NDV and PIV5 HN can adopt various conformations relative to each other and the stalk [16], [38], [19]. It is therefore likely that HN head domains can adopt different conformations on the virion surface as well, and these structural rearrangements may be linked to fusion activation. For MeV H it has been proposed that conversion between two head arrangements, form I and II, is coupled to fusion activation [39]. Based on available structural and functional data, we proposed a potentially general model for paramyxovirus fusion activation in which the head domains of the receptor-binding protein move from the “down position” to the “up position” upon receptor binding to expose residues in the stalk that are the trigger of F protein activation [38]. However, outside of the NDV HN ectodomain structure, no structural information on potential head-stalk interactions has been available for other members of the paramyxovirus family that could support the generality of this model.

Here, we report the structure of the PIV5 HN ectodomain (residues 61–565), which adopts a hybrid state compared to the previously observed 4-heads-down and 4-heads-up conformations. The PIV5 HN heads in the down position form an analogous interaction with the 4HB stalk as observed in the NDV HN ectodomain structure [16], indicating that the formation of this interaction is common to HN proteins of different viruses. The two other PIV5 heads in this structure adopt a “heads up” conformation with one subunit exhibiting a fully helical extension to the stalk 4HB, consistent with a composite structural model (Fig. 1B) of this conformation. A novel DOD interface is observed in the hybrid state and the ability of the full-length HN to form this DOD arrangement is supported by the ability of engineered Cys mutations to form disulfide bonds. As a hybrid between the 4-heads-up and 4-heads-down conformations, the PIV5 structure is consistent with a dynamic head-stalk interaction, in which neuraminidase domain dimers form mobile structural units flexibly linked to the 4HB stalk. Overall, these results demonstrate that two different paramyxovirus HN proteins, from NDV and PIV5, can adopt a conformational state in which the receptor-binding head domains interact with and obscure stalk residues implicated in F protein activation, supporting the hypothesis that this represents a general regulatory feature of the HN-dependent paramyxovirus entry mechanism, potentially applicable to the broader paramyxovirus family as well.

## Results

### PIV5 HN ectodomain crystallized with sialyllactose adopts a 2-heads-up/2-heads-down conformation

The expression construct used previously to determine the X-ray crystal structures of PIV5 HN heads contained the entire ectodomain including the full-length stalk (residues 37–565). However, it has been noted that protease cleavage occurs between residues 55 and 56 of the PIV5 HN ectodomain when expressed in baculovirus infected insect cell culture [19]. Therefore, to boost protein purity and yield in this expression system, a construct comprising residues 56–565 of the PIV5 HN ectodomain was used in this study.

HN was purified, concentrated, and mixed with a slight molar excess of sialyllactose immediately prior to setting up crystallization trials. Upon optimizing conditions, HN crystallized in the I4 space group and diffracted X-rays to ∼2.5 angstroms. The structure was solved by molecular replacement using PIV5 HN structures of the head domain and isolated stalk 4HB. Crystallographic data and refinement statistics are summarized in Table 1.

**Table 1:** Crystallographic data and refinement statistics: PIV5 HN ectodomain.

Data collection	
Source	Advanced Photon Source
Wavelength (Å)	0.97872
Spacegroup	I4
Unit-cell parameters	
a (Å)	194.39
b (Å)	194.39
c (Å)	185.98
α (°)	90
β (°)	90
γ (°)	90
Resolution range (Å)	35.0-2.50 (2.59-2.50)
R_merge_ (%)	8.2 (52.2)
I/σ(I)	20.3 (3.72)
Completeness (%)	100 (100)
Redundancy	6.3 (6.1)

Refinement	
Resolution (Å)	34.4-2.5
No. used reflections (work/free)	116080/1972
R factor/R_free_ (%)	16.6/20.7
No. of residues/atoms	1987/15178
Average B-factors (Å^2^)	50.3
R.m.s. deviations in bond lengths (Å)	0.012
R.m.s. deviations in bond angles (°)	1.18
Ramachandran plot statistics	
Residues in preferred regions (%)	94.9
Residues in allowed regions (%)	4.5

The positions of the head domains relative to the stalk in this structure represent a novel configuration for a paramyxovirus attachment protein, that is a hybrid of the 4-heads-down [16] and 4-heads-up [17] conformations (Fig. 1C–E). Two disulfide-linked heads are in the up position, while two are in the down position (2-heads-up/2-heads-down), and the structure exhibits a novel non-covalent DOD interface between one head in the up position and one head in the down position (hereafter, the DOD interfaces observed in the 4-heads-up and 2-heads-up/2-heads-down conformations will be referred to as DOD_1_ and DOD_2_, respectively) (Figs. 1B–C, S1). Additionally, an interface is observed between residues in two helices on one side of the stalk and one head in the down position similar to that in the NDV ectodomain structure (Fig. 1C). Although sialylactose was included in the crystallization experiments, we do not have any direct structural evidence that its inclusion induced the conformational arrangement observed.

**Figure 2:**
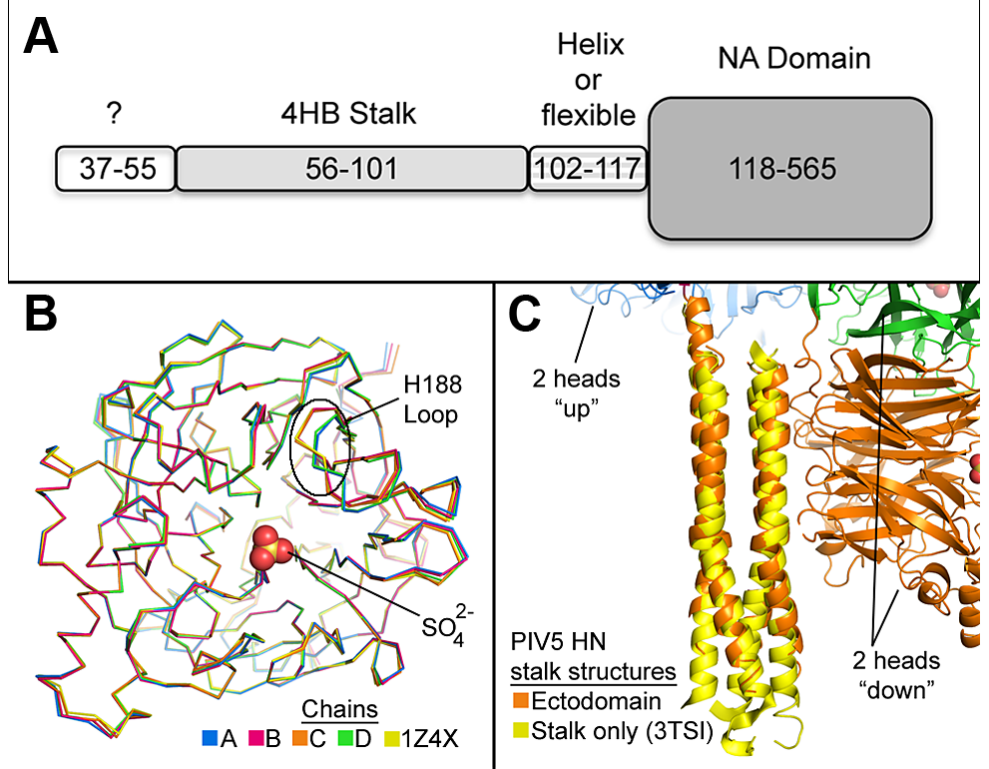
Domains of the PIV5-HN ectodomain. A) Regions of the PIV5-HN ectodomain by residue number. The known structure(s) of each segment are noted. B) Overlay of the head domains of chains A–D (colored blue, magenta, orange, and green, respectively) of the PIV5-HN ectodomain structure and the 1Z4X structure (yellow). The ordered sulfate in the active site is shown as orange and red spheres. The flexible loop containing His 188 is outlined. C) Overlay of the 4HB stalk of the PIV5-HN ectodomain structure (orange) with the isolated PIV5-HN stalk structure (PDB ID: 3TSI, yellow).

Contiguous density linking a stalk helix and head domain is also observed (Fig. 1F). The stalk of chain B extends beyond the 4HB core, which ends at residue L101, forming an extended alpha helix through N110. This extended helix also corresponds to the positioning of the head of this chain higher than its covalent partner and is consistent with the 4-heads-up conformation, where one head of each covalent dimer extends higher than its partner leading to a ∼30° offset between covalent dimers across the DOD_1_ interface. Of note, although D105 and E108 of chain B are within an extended helix, they project toward the central 4HB axis and may disrupt the coiled coil from propagating beyond L101 (Fig. 1F). Accordingly, residues 102–110 of chain A/E form a non-structured linker (residues 103–106 are disordered). Interestingly, chains A and B cross at C111 where they form a disulfide bond and short antiparallel β-sheet. Residues 113–117 form a partially helical strand connecting with the head domain at I118. Therefore, residues 56–101 form the 4HB stalk that is followed by a linker region (residues 102–117), part of which can extend from the 4HB stalk as an isolated helix (residues 103–110), that eventually connects to the globular head domain at I118 (Fig. 2A).

Each of the four globular head domains overlays with good agreement with the search model (PDB ID: 1Z4X) (≤0.210 RMSD over ∼404 atoms, Fig. 2B). The only notable exception is the position of the active site loop containing H188 and the conserved D187 in the two heads that form the novel DOD interface (discussed below). Electron density was also observed for the stalk of HN. The final model aligns well with the previously solved isolated PIV5 HN stalk structure (residues 56–105, 0.627 RMSD over 143 atoms) [21] including a relatively non-supercoiled region in the upper portion of the stalk comprised of an 11-mer repeat that transitions to a supercoiled region comprised of a heptad repeat in the lower half of the observed stalk (Fig. 2C).

### A novel dimer-of-dimers interface

**Figure 3:**
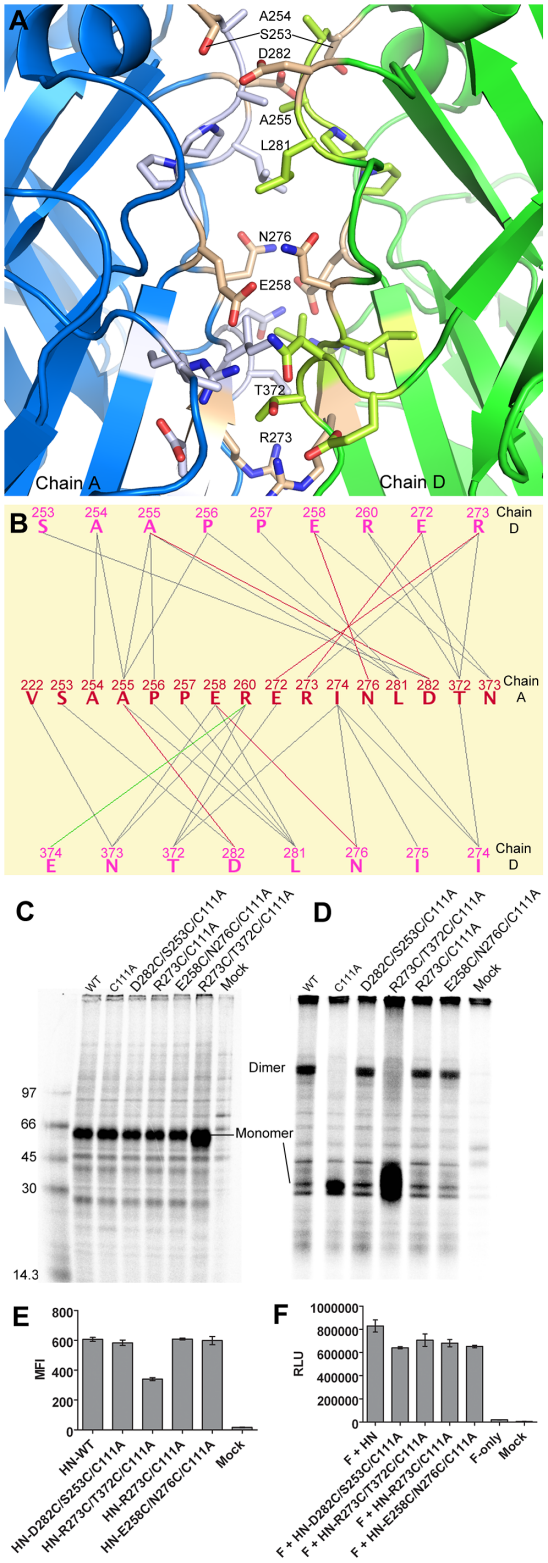
A novel PIV5-HN head domain dimer-of-dimers interface. A) Cartoon representation of the PIV5-HN ectodomain structure dimer-of-dimers interface (DOD_2_). Chains A and D are blue and green, respectively. Residues comprising the interface are shown as lighter color sticks, and residues that form disulfide bonds are colored tan. B) Contact map showing specific interactions within the interface. Gray, green, and red lines represent hydrophobic, electrostatic, and hydrogen bond interactions, respectively. Figure produced using the MONSTER server for analysis of macromolecular complexes [52]. C–D) Reducing and non-reducing SDS-PAGE gels, respectively, showing HN Cys mutants immunoprecipitated from cell extracts. The positions of monomer and dimer are indicated. The C111A mutation eliminates wt dimer formation. E) Flow cytometry reveals that each of the HN Cys mutants were expressed on the surface of cells within 2-fold of wt. MFI = mean fluorescence intensity. F) A cell-cell fusion assay indicates each of the HN Cys mutants remained fusogenic at levels similar to wt. Experiments were done in triplicate and error bars represent S.E.M in E–F.

A novel dimer-of-dimers interface lacking pseudo-tetrameric symmetry is observed between one head of one disulfide-linked dimer in the up position (chain A) and another (chain D) in the down position (DOD_2_, Fig. 1C, 3A). This ∼2-fold symmetric interface buries ∼1,139 Å^2^ (total) of solvent-accessible surface area and includes 16–17 residues from each of the two heads. The interface is generally hydrophobic in nature with 31 hydrophobic interactions and seven electrostatic interactions including six hydrogen bonds (Fig. 3B). For comparison, DOD_2_ is larger than DOD_1_ observed in the 4-heads-up conformation, which has a total buried surface area of ∼846 Å^2^ (using similar methodology). However DOD_2_ is much smaller than the dimer interface (∼3919 Å^2^, chains A/B), which is slightly expanded here compared to that previously observed due to the more complete model.

We probed the functional significance of this interface by introducing cysteine mutations into a mammalian expression construct of HN containing a C111A mutation that eliminates wt covalent dimer formation. Reducing and non-reducing SDS-PAGE analysis following immunoprecipitation of radiolabeled protein revealed that the R273C point mutation and mutant pairs D282C/S253C and E258C/N276C are capable of extensive disulfide bond formation manifested as dimer bands on non-reducing SDS-PAGE gels (Fig. 3C–D). At the DOD_2_ interface, R273 residues have a Cα-Cα distance of 7.14 Å, D282/S253 residues have a Cα-Cα distance of 6.63 Å and E258/N276 residues have a Cα-Cα distance of 7.44 Å, consistent with the observed disulfide bond formation. Due to the lack of 4-fold symmetry in the head domains, the two cysteine mutations not paired in a disulfide bond at DOD_2_ can potentially form disulfide bonds with neighboring HN tetramers leading to higher order covalent structures. Indeed, higher molecular weight species are observed at the top of the non-reducing gel. However, this band is also observed in the mock-transfected lane making it unlikely that these species are HN (Fig. 3D). Although the R273C single mutant is capable of disulfide bond formation, no dimer is observed when R273C is combined with T372C despite overexpression of this double mutant compared to wt. This result is likely due to a preference for intrachain vs interchain disulfide bond formation, as these residues are also adjacent in the monomer, with an intrachain Cα-Cα distance of 7.2 Å.

Interestingly, HN mutations harboring the successfully engineered disulfide bonds are each expressed on the surface of cells at levels similar to wt, and they are fusogenic at 75–85% wt levels in a cell-cell fusion assay (Fig. 3E–F). Finally, we note that while DOD_2_ is formed within individual tetramers in this structure, a crystal packing interface equivalent to DOD_1_ is formed between tetramers in the crystal lattice. In the original crystal structure of PIV5 HN [17], we noted that the DOD_1_ packing interactions between dimers must occur both within and between tetramers, forming an extended ribbon throughout the crystal lattice. In the current crystal form, the DOD_1_ packing interactions between tetramers further points to the favorable nature of this interface. The possible significance of this observation is discussed further in supporting information (Text S1 and Fig. S2).

### The stalk/head interface

**Figure 4:**
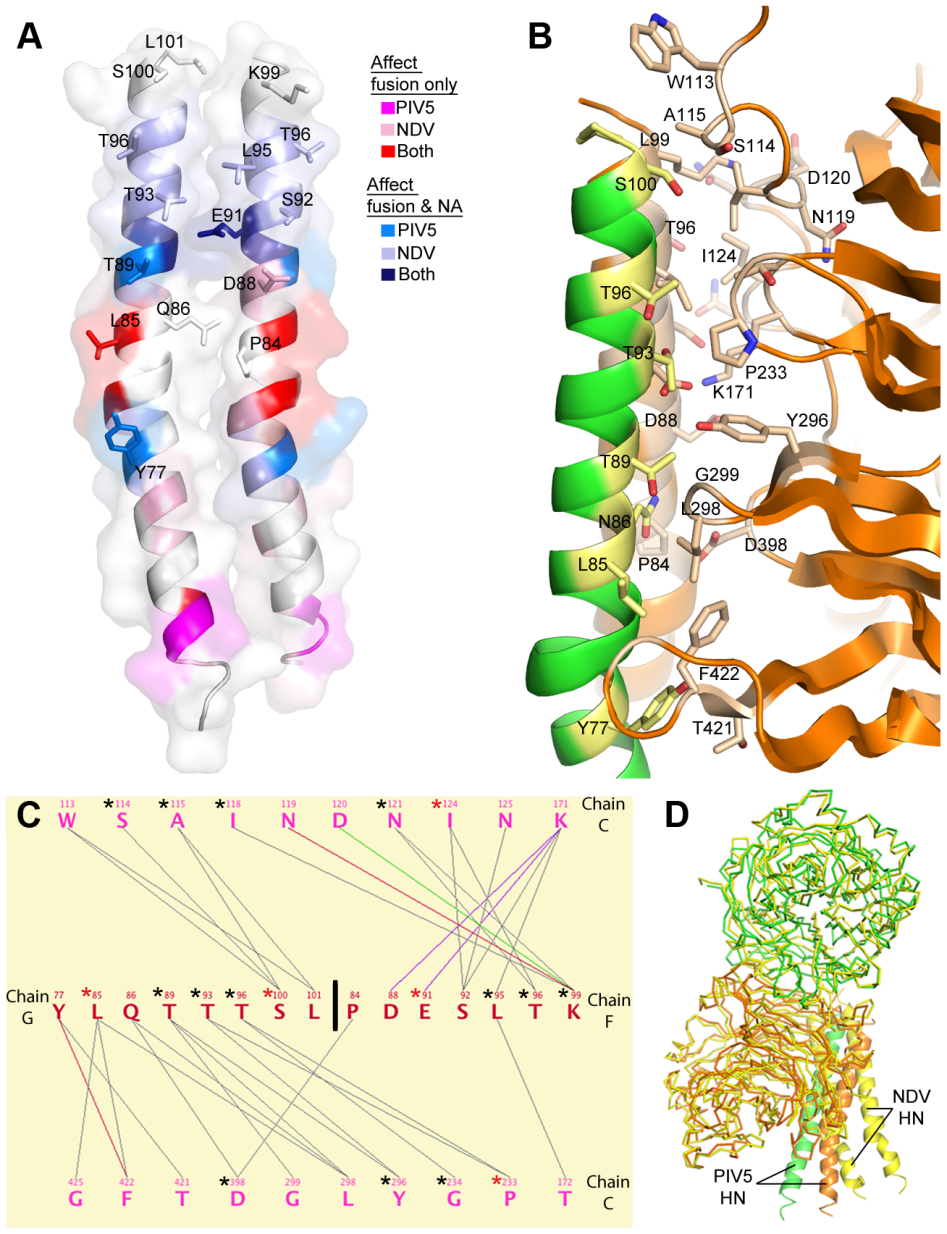
Stalk/head domain interface in the PIV5-HN ectodomain structure. A) Cartoon rendering with transparent surface of the PIV5-HN stalk showing the locations of known category I (affect fusion only) and category II (affect fusion and NA) mutations in NDV and PIV5 HN (adapted from [21] and expanded to include current data). The stalk residues at the stalk/head interface are shown as sticks and labeled. B) Zoomed-in view of the stalk/head interface with individual chains colored as in Fig. 1. Sidechains at the interface are shown as lighter colored sticks. C) A MONSTER contact map of the DOD_1_ interface analogous to that in Fig. 3B. PIV5 residues at the stalk/head interface that align with residues present in the NDV stalk/head interface are highlighted with an asterisk. Red asterisks mark identical residues across both interfaces. D) Overlay of the head domains of a covalent dimer of NDV HN (yellow) with PIV5 HN (green and orange) showing the different angles of the stalks relative to the heads in the two structures.

The recent crystal structure of the NDV HN ectodomain revealed an interface between head domains and the stalk (Fig. 1A), which also overlaps a stalk surface implicated in direct interactions with F. Interestingly, mutations in a corresponding region of the PIV5 HN stalk also affect fusion and neuraminidase activities (Fig. 4A), and it has been predicted that PIV5 HN would form a similar head-stalk interaction [21]. A stalk/head interface is indeed observed in the current structure, which resembles that of NDV HN in that covalently-linked heads are in the down position allowing one head to form a similarly sized interface with the stalk (buried solvent-accessible surface area = 1,294 and 1,185 Å^2^ for PIV5 and NDV using similar methods, respectively) (Fig. 4B–C). Overall, there are 15 residues from two stalk helices and 20 residues from the head that form the interface. Like the stalk/head interface in NDV HN, the PIV5 HN interface is primarily hydrophobic with 28 hydrophobic interactions and 5 electrostatic interactions including two hydrogen bonds and two salt bridges. While 18 of the residues that comprise this interface in PIV5 and NDV HN align based on primary sequence alignment, none of the interactions between these residues are identical (Fig. 4C). Additionally, the angle of the heads relative to the stalk differs between the two molecules by ∼30° (Fig. 4D).

### Point mutations at the stalk/head interface

**Figure 5:**
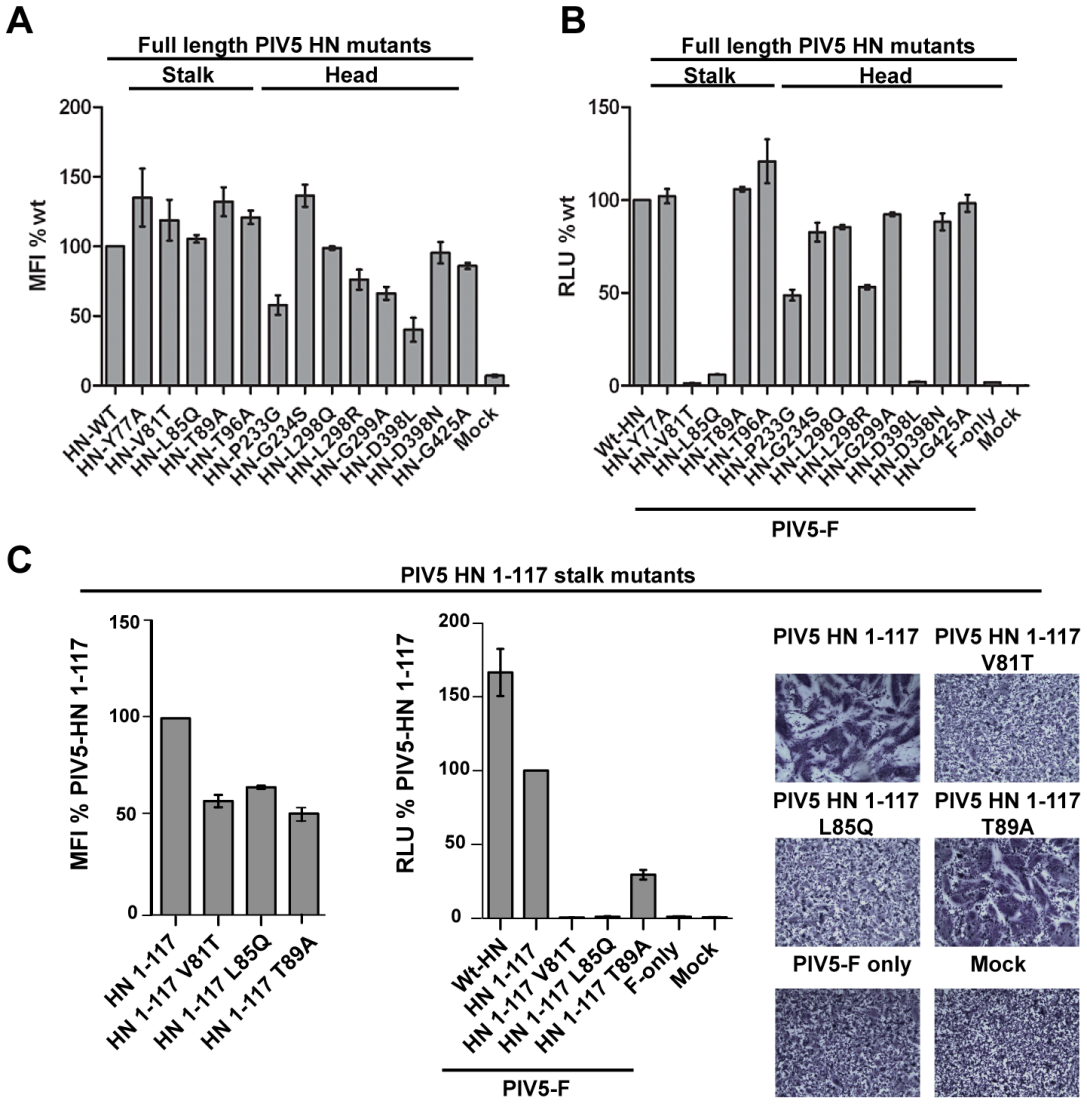
Surface expression and cell-cell fusion data of point mutations in the PIV5-HN stalk/head interface. A) Flow cytometry reveals that each of the HN mutants were expressed on the surface of cells within ∼2-fold of wt. MFI = mean fluorescence intensity. B) The cell-cell fusion assay indicates V81T and L85Q mutations block fusion, whereas the other mutations fuse at levels similar to wt. C) Mutations V81 and L85Q were introduced into the PIV5 HN stalk and the mutant stalks co-expressed with F. Left panel: Surface expression of PIV5 HN stalk mutants; middle panel: reporter assay for fusion; right panel: syncytia formation in BHK-21 cells. RLU = relative luminescence units. Experiments were done in triplicate and error bars represent S.E.M in A–C.

To investigate if mutations at or near the stalk/head interface would affect fusion, we made single point mutations of residues lying within both the stalk and head domains. Flow cytometry revealed that the point mutants were expressed at the cell surface at levels within ∼2-fold of wt (Fig. 5A), except for the D398L mutation. Within the stalk region, only V81T and L85Q were significantly impaired for cell-cell fusion (Fig. 5B), while mutations Y77A, T89A and T96A were fusion competent. Interestingly, Y77A is fusion active whereas a mutation that introduces a glycan at this position (N77 mut) is not [21]. Of the eight mutants within the head domain, only one (D398L) showed reduced fusion activity, but this also corresponded to a significant reduction in D398L expression levels. The mutational data further support the significance of this stalk region in F activation and fusion, given the functional effects of the V81T and L85Q mutations. As the headless PIV5 HN stalk is active in fusion, mutations of the head domain that disrupt the head-stalk interaction may not be defective in fusion activation, but may mimic the headless HN stalk activity. To investigate whether the V81T and L85Q mutations affect F activation directly, these were introduced into the headless PIV5 HN stalk construct and examined. Even though surface expression for the mutant stalks was reduced only ∼50%, fusion promotion was completely inhibited (Fig. 5C). Thus, these data suggest that the V81T and L85 mutations cause direct interference with the HN stalk-F interface and do not reduce fusion by affecting HN head-stalk mechanics.

### Movement of H188 is correlated with changes in electron density for ligand at the active site in head domains at the DOD_2_ interface

**Figure 6:**
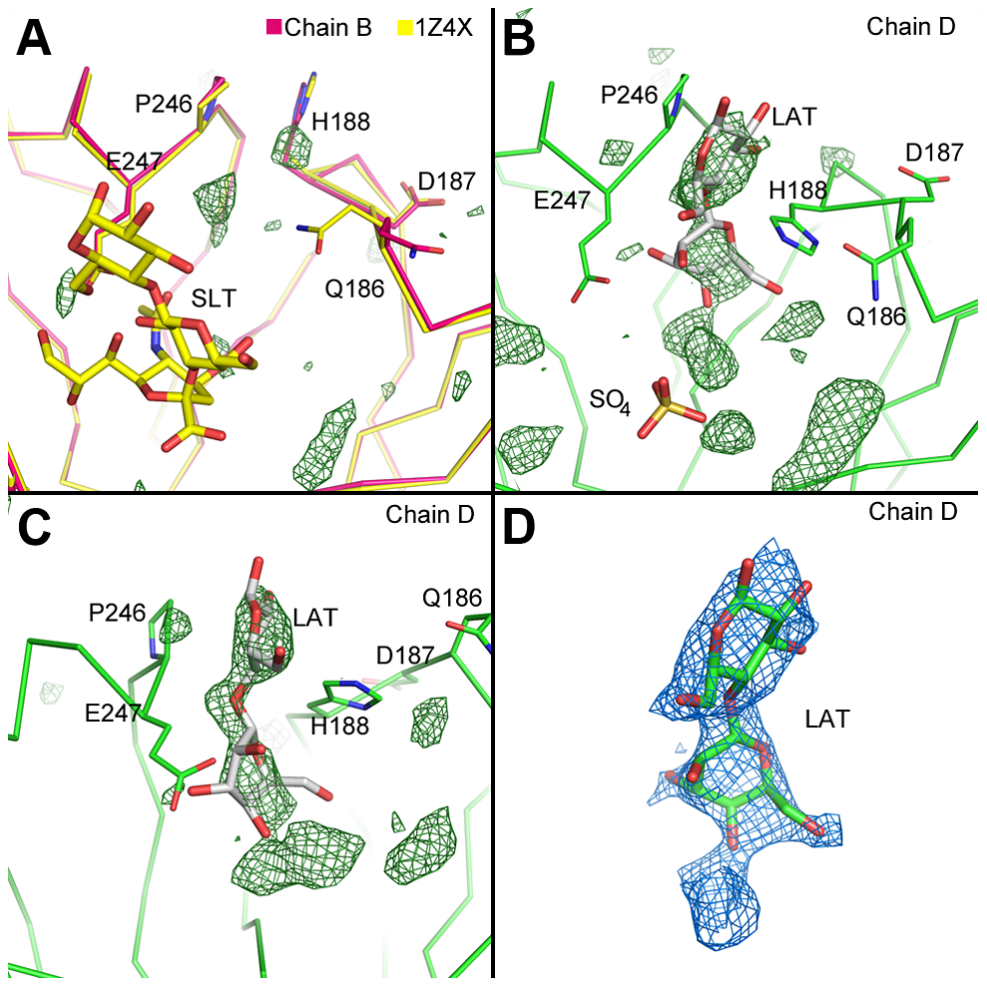
Electron density at the PIV5-HN ectodomain active sites. A) Close-up view of the active site of chain B (magenta) overlaid with the 1Z4X structure (yellow). The sulfate ion in chain B is not shown, but sialyllactose (SLT) from 1Z4X is shown as sticks. The sigma-A weighted Fo-Fc map rendered at 3.8 sigma is shown as green in A–C. B) Similar view as in A but of chain D. Lactose (LAT, gray) is modeled into significant positive difference density near H188 that is found in chains A and D but not in chains B and C. The sulfate ion is shown as sticks. C) Another view of the scene in B. D) Sigma-A weighted 2Fo-Fc map rendered at 1.0 sigma resulting from refinement of lactose fit into the positive difference density in chain D (orientation as shown in B).

The previous PIV5 HN crystal structures of the head domain were solved using various conditions including native, receptor-bound (sialic acid and sialyllactose), and inhibitor-bound (DANA) at either pH 7.0 or 8.0 [17]. There was good agreement between the structures, and no conformational change was observed upon sialyllactose or DANA binding (sialic acid could be converted to DANA by HN as observed with other neuraminidases). However, these ligand-bound structures were obtained by soaking soluble ligands into native crystals, and preformed crystal contacts may prevent movement that might otherwise occur. As mentioned above, each of the HN heads in the current structure superpose with good agreement with the previously solved structures (Fig. 2B). However, a notable exception is the position of the loop containing H188 in chains A and D that interact to form the DOD_2_ interface. In these chains, H188 points directly into the active site whereas in the other heads of this structure, and previously solved structures, H188 is pointing up and away from the active site (Fig. 6A–B). This loop has been observed to be flexible as residues 186/7-190 were disordered in two of the previous PIV5 HN head structures. Furthermore, this loop has been observed in unique conformations in a low-pH NDV Kansas HN and NDV Ulster HN crystal structures [12], [40].

Interestingly, the movement of H188 in the current structure is correlated with electron density observed in the active site of each chain. In the active site of the chains not involved in the DOD_2_ interface (chains B and C, Fig. 6A), there was no interpretable electron density except for spherical density that was modeled as a sulfate ion coordinated by R405, R495, and Y523. However, in the heads of the DOD_2_ interface (chains A and D) additional electron density was observed near the alternately oriented H188. Part of this density appears consistent with the lactose moiety of α(2,3′)-sialyllactose potentially generated during crystallization, given the enzymatically permissive pH (pH = 6.6) of the crystallization conditions (Fig. 6B–D). However, the crystallization buffer includes ∼0.5 M ammonium sulfate, and high salt concentrations are known to inhibit NA activity [2]. Fitting lactose to the density clearly does not satisfy the density that protrudes deepest into the active site (Fig. 6B–D); however, significant negative difference density is associated with modeling the ‘sialic acid’ portion of α(2,3′)-sialyllactose into this density (not shown). Due to the ambiguity associated with ligand identification and conformation, the final model does not include atoms fit to this active-site density.

## Discussion

Here we observe that the PIV5 HN ectodomain can form a head-stalk interaction similar to the arrangement in the structure of the NDV HN [16], providing further evidence that this conformational state could regulate F activation across different members of the paramyxovirus family. We further observed that the PIV5 HN protein can adopt a hybrid conformation consisting of 2-heads-down and 2-heads-up, with pairs of dimeric heads moving as unified structural units. This hybrid conformational state demonstrates that the PIV5 HN tetramer has inherent flexibility and potential for asymmetry in the head region dimer-of-dimers, consistent with the possibility that sialic acid receptor binding could reorient head domain dimers, exposing the stalk region for F engagement and activation. This hybrid structure also provides a concrete model for the fully helical head-stalk connection in the hypothesized 4-heads-up conformational state.

The PIV5 HN ectodomain structure identifies a novel dimer-of-dimer interface, DOD_2_, which is readily formed in the intact HN, as revealed by cysteine mutagenesis and disulfide bond formation. Engineered disulfide bonds bridging the PIV5 HN DOD_2_ interface formed readily, consistent with the ability of the NA domains to access the observed 2-heads-up/2-heads-down arrangement in intact HN. Interestingly, covalent linkage at the DOD_2_ interface does not impair HN's ability to activate fusion. It would not be possible for all of the heads to simultaneously be in the down or up positions when the heads are crosslinked via this interface, yet all of the mutants that form disulfide bridges are fusion competent. These results suggest that either exposure of one stalk site could result in F activation, or alternatively that exposure of both stalk sites, but not full formation of a 4-heads-up conformation, is sufficient for F activation. The mutant proteins may be active in the observed 2-heads-up/2-heads-down hybrid state or may be able to adopt a state where none of the heads engage the stalk after receptor binding, thereby activating F. Movement of one pair of heads to the up position may be sufficient to trigger fusion, as recent data suggests for measles virus H activation [41]. The cysteine mutants provide further evidence for the dynamic and flexible nature of HN head arrangements.

An unexpected observation from the current structure is a conformational change within the active site of the two HN heads comprising the DOD_2_ interface.

Early crystal structures of NDV and hPIV3 revealed a pliable active site within HN monomers that could switch between sialic acid binding and catalytic activity, however, no conformational changes were observed in the hPIV3 structure beyond the active site upon ligand binding [12], [14]. In addition, no structural changes were observed in PIV5 HN comparing the non-liganded, inhibited, and receptor bound structures [17]. However, in the current structure, the position of the loop containing H188 is altered in the chains that form the DOD_2_ interface, with the H188 side chain pointing into the active site, as compared to the other chains and previously determined HN crystal structures. Electron density is observed near H188 in this altered conformation, which appears consistent with the binding of a partially disordered carbohydrate moiety at an alternate active site location also not previously observed. The unique conformation of H188 suggests that it might play a role in receptor hydrolysis or in facilitating alternative receptor binding modes. The H188 loop is directly adjacent to the loop containing residues 221–223 at the DOD_2_ interface. Small movements of this loop within chains A and D compared to B and C may indicate that subtle movements caused by formation of DOD_2_ could impact active site residues (or vice versa). Local chemical environments within the crystals, NA domain conformations and/or dynamics within the lattice and other parameters may play a role in the visualization of this extra density within only two of the active sites.

**Figure 7:**
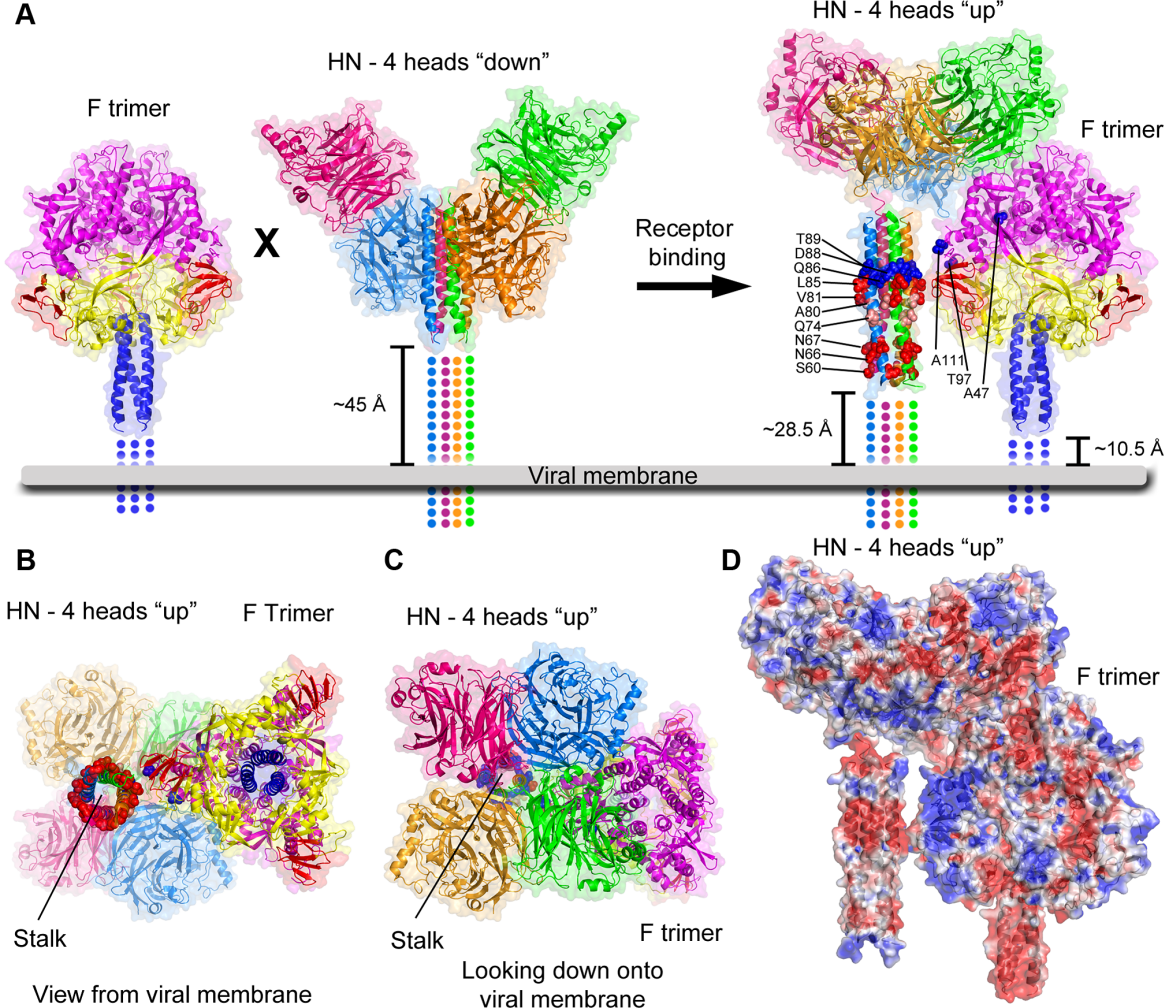
Model of paramyxovirus activation of F by HN. The heads of HN in the 4-heads down conformation block F activation by preventing access to critical residues in the mid-upper HN 4HB stalk. However, upon receptor binding HN heads move to the up position revealing residues in the HN stalk that interact with and trigger F for fusion. HN molecules were aligned by relevant domains with the 2-heads-up/2-heads-down structure to provide proper orientation of the 4-heads-up and 4-heads-down conformations with respect to one another and the exact spacing of the heads relative to the stalk in the 4-heads-up model. The distances from the membrane that missing residues from the F and HN ectodomain crystal structures would occupy if they are fully helical are indicated. Without invoking flexibility of the membrane proximal portion of the HN ectodomain, a non-canonical transmembrane domain region for F [53], or rearrangement of surface loops in F or HN heads - the spacing predicts limited ways in which F can contact the stalk of HN. PIV5 HN-stalk residues (red) or equivalent residues identified in MeV H (blue) and NDV HN (light pink) implicated in direct contact with F are shown as spheres. Approximately equivalent PIV5 F residues as those identified in canine distemper virus implicated in direct contact with MeV H are shown as spheres (blue). Missing portions of the ectodomain structures are shown as dotted lines, and the viral membrane is shown as a thick gray line. B) View of the F/HN interaction model shown from the bottom (view from the viral membrane) and C) top (looking down onto the viral membrane). In A–C, HN is colored as in figure 1. The cleaved form of PIV5 F (PDB ID: 4GIP) is shown. Domains I, II, III, and HRB are colored yellow, red, magenta, and blue, respectively. D) Electrostatic surface map of the F/HN interaction. The view is the same as in A above. Surfaces were calculated using the Adaptive Poisson-Bolzmann Solver [54] and PDB2PQR software (v1.3) [55], [56] via the AMBER algorithm within PyMOL. The electrostatic potential ranges from −2 (red) to +2 (blue) kT/e.

Contiguous electron density linking the stalk and head of one HN subunit in the current PIV5 HN structure supports the possibility that HN can adopt the modeled 4-heads-up conformation, where the 4HB stalk is located in the center and directly beneath the four heads (Fig. 1B, S1). The current structure establishes the height of the heads relative to the stalk in the 4-heads-up model, assuming that the negative charge from D105 and E108 side chains can be accommodated in two opposing helical extensions beyond the 4HB. If the membrane proximal portions of the HN and prefusion F protein stalks, for which structural information is not available, are assumed to be fully helical, modeling of the F/HN interaction with HN in the 4-heads-up conformation suggests that steric restrictions could still affect F/HN interactions. For example, F access might be limited to fully extended heads (‘defined’ by the helix extension of residues 103–110, Fig. 1D) and to the more open gap between dimers (Fig. 7A–C). The ∼30° tilt between dimers in the 4 heads up model lowers the height of two of the heads, potentially blocking F interactions from these angles (Fig. 1D), unless the N-terminal region of the stalk is flexible and can extend further from the membrane surface. In the absence of such stalk extension, steric restrictions could limit the stoichiometry of F trimer to HN tetramers to 2∶1 or 1∶1 interactions. Steric access restrictions would explain why HN would not activate F in the 4-heads-down conformation, where two sides of the stalk are at least partially accessible.

This HN-F model also spatially aligns corresponding residues identified in MeV H implicated in direct F contacts (Fig. 7A) [37]. Residues in the PIV5- and NDV-HN stalk implicated in direct interaction with F also map to approximately the right height for the contact point assumed in this model [37]. Curiously, mutations in the PIV5 HN stalk that are exposed and disrupt only fusion map to a lower regions of the 4HB. This may be because the PIV5 HN stalk mutations introduced carbohydrate chains [38] and the mutations may block fusion activation by steric hindrance, disrupting F-interactions from a distance. Single point mutations V81T and L85Q narrow down the putative PIV5-HN interacting region (Fig. 5) and this region for PIV5-HN aligns well with the analogous region proposed for NDV-HN and measles virus-H [37] [16].

Electrostatic surface maps also provide additional support for the general features of this HN-F model. A band of negative charge in the HN stalk that corresponds to the presumed F-interaction region aligns with charged regions of F (Fig. 7D). Finally, the model suggests why the glycoprotein spikes of paramyxoviruses appear to be approximately the same height (“parallel-head” model) [42] in electron micrographs [1] versus the “staggered-head” model [27], [37] that fits better with available biochemical and structural data (i.e., F is as tall as HN when HN is in the 4-heads-down conformation, but HN is taller in the 4-heads-up conformation).

Much biochemical and mutagenesis evidence spanning a variety of paramyxoviruses has established the HN stalk as the key site for triggering F, but with potentially conflicting mechanistic data on the structures and role of the receptor-binding head domains. Experiments involving disulfide crosslinking of MeV H head dimers have indicated that movement between heads across the dimer interface may be important for fusion [43]. However, a similar analysis of head dimers in NDV and hPIV3 indicated that movement at the dimer interface is not required [44]. It has been proposed that subtle changes at the head dimer interface may trigger larger changes, perhaps propagating to a dimer-of-dimers interface [45]. Different crystal forms of MeV H suggested that rearrangement of dimers in the tetramer could possibly trigger F by also affecting the 4HB stalk [39]. Disulfide bond stabilization of the central region of the morbillivirus H protein stalk is inhibitory, and fusion is recovered upon reduction of the disulfides [23], [41], [26]. These authors suggest an unwinding of the supercoiled region of the stalk may activate F. This model shares several features in common with a membrane-proximal stalk-extension model that we have discussed previously [16]. Although morbillivirus and henipavirus entry mechanisms may differ from other paramyxoviruses, potentially in the strength and timing of H/G and F interactions, a role for the attachment protein stalk regions in F activation is a common underlying feature. We suggest that inhibitory head-stalk domain interactions across the broader paramyxovirus family could occur, providing a unifying model for regulating F activation. For morbillivirus and henipavirus family members, receptor-dependent alterations in head-stalk interactions could allow F full engagement of the stalk site necessary for fusion activation, despite the potential formation of pre-fusion H/G and F complexes. Additional experiments are necessary to establish whether morbillivirus and henipavirus entry mechanisms share the key elements of this stalk-exposure model.

In summary, while many specific details of the F/HN interaction remain to be elucidated, the present structure provides further evidence that paramyxovirus HN head domains interact with the 4HB stalk and that movement from a ‘heads-down’ to a ‘heads-up’ conformation allows F access to critical fusion-promoting residues in the stalk. Notably, this ‘stalk-exposure’ model fits with the “provocateur” mechanism of fusion protein activation [46], rather than a “clamp model” (reviewed in [47]) consistent with available data from paramyxoviruses that utilize HN attachment proteins. It remains possible that other paramyxoviruses promote fusion differently, although the common role of the attachment glycoprotein stalk domain in F activation indicates that additional mechanistic similarities will be conserved across the virus family.

## Materials and Methods

### Cells, plasmids, and antibodies

Vero cells and 293T cells were maintained in Dulbecco's modified Eagle medium (DMEM) supplemented with 10% fetal bovine serum (FBS). BHK-21F cells were grown in DMEM containing 10% FBS and 10% tryptose phosphate broth. BSR-T7/5 cells were grown in DMEM containing 10% FBS, with 500 µg/ml G418 added every third passage. Hi5 insect cells were maintained in Express 5 serum free medium (Gibco) supplemented with 10% GlutaMax (Gibco) and Sf9 insect cell lines (for generating baculovirus stocks) were maintained in SF900 II medium containing 10% FBS. The construct for PIV5 HN ectodomain (56–565) expression has been described previously [19]. Antibody specific for HN was polyclonal antibody (PAb) R471, raised in rabbits against the purified HN ectodomain expressed by a recombinant baculovirus in insect cells.

### Protein expression and purification

Hi5 insect cells were infected (moi = 2) with a recombinant baculovirus stock containing the PIV5-HN ectodomain (56–565) construct and harvested 65 hr post infection. Protein was purified from the supernatant by affinity chromatography using Ni-NTA agarose (Qiagen) and was >90% pure by SDS-PAGE and Coomassie brilliant blue staining analysis. The S- and His-tags were cleaved from the expressed protein as previously described [21].

### Crystallization

Purified protein was buffer exchanged into 10 mM Tris pH 7.4, 50 mM NaCl and concentrated to ∼10 mg/mL, and a 1.2 molar excess of sialyllactose (Sigma) to HN monomers was added immediately prior to setting up crystallization trials. Initial crystals were obtained with the Crystal Screen HT (Hampton) by the hanging drop vapor diffusion method using a Mosquito (TTP LabTech) at the High Throughput Analysis Lab (Northwestern University, Evanston). After optimization, crystals were grown at room temperature by the sitting drop vapor diffusion method over a reservoir solution containing 1.6 M ammonium sulfate, 0.1 M MES monohydrate pH 6.5, 10% v/v 1,4-dioxane. Drops consisted of protein and precipitant at a 2∶1 ratio. The crystals were flash frozen in liquid nitrogen using the crystallization condition diluted with glycerol to 20% as the cryoprotectant solution.

### Data collection, structure determination and refinement

A native dataset was collected at the Life Sciences Collaborative Access Team (LS-CAT) beamline at the Argonne National Laboratory Advanced Photon Source and processed to 2.5 Å using HKL2000 [48]. The monomeric neuraminidase (PDB ID: 1Z4X) and 4HB stalk domains (PDB ID: 3TSI) of PIV5 HN were used as search models for molecular replacement to determine initial phases in the I4 spacegroup. Four NA domain monomers and the 4HB stalk domain were found in the asymmetric unit. Subsequent model building, structure refinement, and validation were performed with Coot [49], PHENIX Refine [50] and MolProbity [51], respectively. Use of TLS parameters (as recommended by Phenix) and individual B-factors during late stages of refinement helped to lower the Rfree values, however, the use of non-crystallographic symmetry restraints increased Rfree values. The data collection and final refinement statistics are shown in Table 1. The atomic coordinates and structure factors have been deposited in the Protein Data Bank, www.pdb.org (PDB ID: 4JF7).

### Cloning and mutagenesis

A previously described pCAGGS-HN expression construct harboring the PIV5 (W3A) HN gene was used [19]. Mutants in pCAGGS HN were constructed as described previously [21].

### Immunoprecipitation (IP) and SDS PAGE

IP of HN muts from transfected 293T cells was performed as described previously [21].

### Flow cytometry

Surface expression of HN mutants in transfected 293T cells was performed as described previously [21].

### Cell-cell fusion assay

Syncytia formation was measured in BHK-21 cells co-transfected with PIV5 F and HN mutants as described previously [21].

### Luciferase reporter assay

Levels of fusion between Vero cells co-transfected with pCAGGS F, pCAGGS HN, and pT7 luciferase and BSR-T7/5 cells expressing T7 RNA polymerase were quantitated using a previously described Luciferase reporter assay [21].

## Supporting Information

Figure S1 **Top view of the 4-heads-up model.** View of the 4-heads-up model rotated 90° along the horizontal axis relative to Fig. 1B. The view is looking down onto the viral membrane. The dimer and DOD_1_ interfaces observed in the 1Z4X crystal structure are shown. Coloring is as in Fig. 1B.
(TIF)

Figure S2 **Organization of molecules in PIV5 HN crystals.** A) In the previously described PIV5 HN crystal structures lacking density for the N-terminal stalk (e.g., 1Z4X), covalent HN dimers were organized in a repeating array such that any two pairs of dimers (circled) overlays with any other dimer pairs (i.e., the dimer of dimers formed by the A (green) and B (red) dimers overlays with the B and C (blue) dimer pair, each forming the DOD_1_ interface). However, one DOD_1_ interface must define an intramolecular interaction while the other forms an intermolecular interaction. B) In the 2-heads-up/2-heads-down PIV5-HN ectodomain structure, a covalent dimer in the up position from HN tetramer #1 (white) interacts with a covalent dimer in the down position from the adjacent HN tetramer #2 (green). This intermolecular interaction forms the DOD_1_ interface observed between any pair of dimers in the 1Z4X structure (red). A sulfate ion shown as spheres marks the active sites. C) Top down view of a set of four interconnected HN molecules in the 2-heads-up/2-heads-down structure with the intermolecular DOD_1_ and intramolecular DOD_2_ interfaces highlighted by a diamond and oval, respectively.
(TIF)

Text S1 **HN ecto Supporting Info_txt.** Supporting information text S1.
(DOC)
