# Supplementary figures and images for: Structure of the Parainfluenza Virus 5 (PIV5) Hemagglutinin-Neuraminidase (HN) Ectodomain
Source: PLoS Pathog. 2013 Aug 8;9(8):e1003534. doi: 10.1371/journal.ppat.1003534 (PMC3738495; doi:10.1371/journal.ppat.1003534)

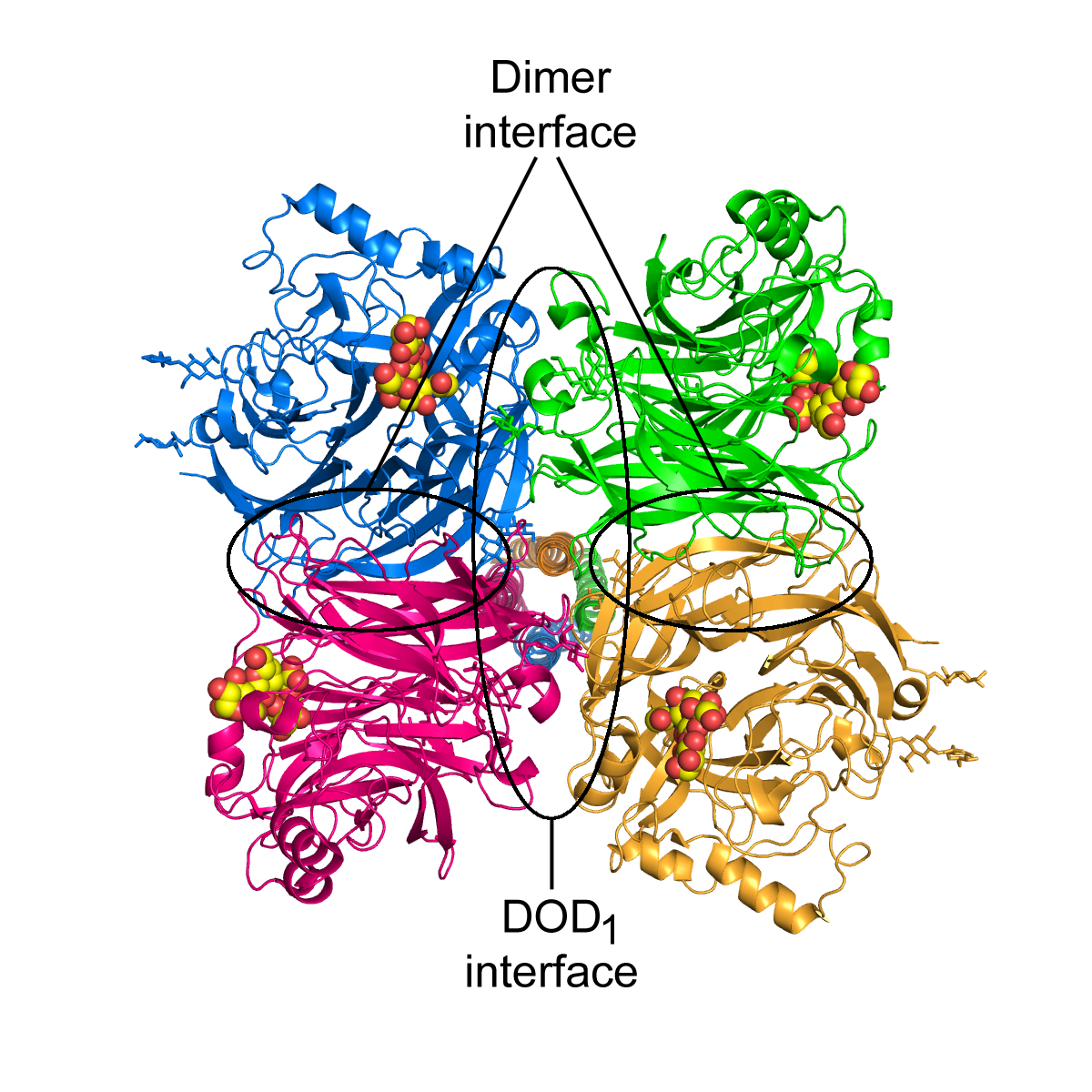

Supplement: Figure S1 — Top view of the 4-heads-up model. View of the 4-heads-up model rotated 90° along the horizontal axis relative to Fig. 1B. The view is looking down onto the viral membrane. The dimer and DOD1 interfaces observed in the 1Z4X crystal structure are shown. Coloring is as in Fig. 1B. (TIF) [file ppat.1003534.s001.tif]

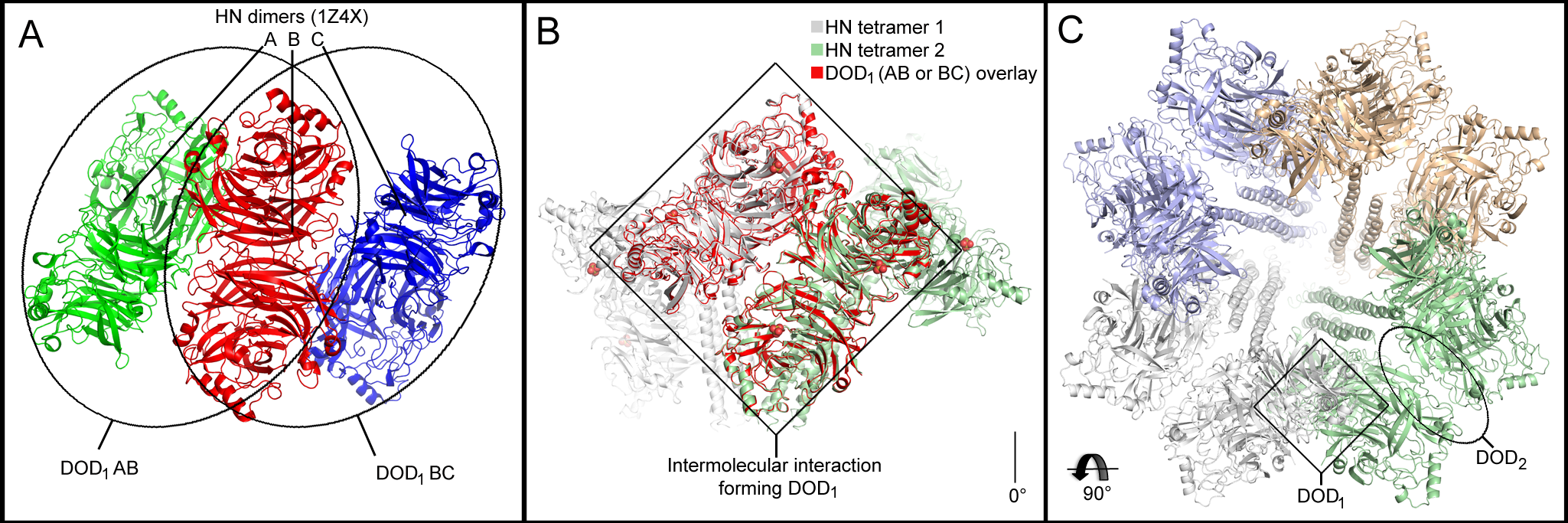

Supplement: Figure S2 — Organization of molecules in PIV5 HN crystals. A) In the previously described PIV5 HN crystal structures lacking density for the N-terminal stalk (e.g., 1Z4X), covalent HN dimers were organized in a repeating array such that any two pairs of dimers (circled) overlays with any other dimer pairs (i.e., the dimer of dimers formed by the A (green) and B (red) dimers overlays with the B and C (blue) dimer pair, each forming the DOD1 interface). However, one DOD1 interface must define an intramolecular interaction while the other forms an intermolecular interaction. B) In the 2-heads-up/2-heads-down PIV5-HN ectodomain structure, a covalent dimer in the up position from HN tetramer #1 (white) interacts with a covalent dimer in the down position from the adjacent HN tetramer #2 (green). This intermolecular interaction forms the DOD1 interface observed between any pair of dimers in the 1Z4X structure (red). A sulfate ion shown as spheres marks the active sites. C) Top down view of a set of four interconnected HN molecules in the 2-heads-up/2-heads-down structure with the intermolecular DOD1 and intramolecular DOD2 interfaces highlighted by a diamond and oval, respectively. (TIF) [file ppat.1003534.s002.tif]
