# Supplementary material for: Structure of the Parainfluenza Virus 5 (PIV5) Hemagglutinin-Neuraminidase (HN) Ectodomain
Source: PLoS Pathog. 2013 Aug 8;9(8):e1003534. doi: 10.1371/journal.ppat.1003534 (PMC3738495; doi:10.1371/journal.ppat.1003534)
Supplement: Text S1 — HN ecto Supporting Info_txt. Supporting information text S1. (DOC) [file ppat.1003534.s003.doc]

**SUPPORTING INFORMATION**

**Text S1:** The arrangement of PIV5 HN molecules in the two different crystal forms of PIV5 HN is noteworthy. In the "4-heads-up crystals", HN heads were organized as an extended array of dimers interacting through the DOD_1_ interface . Because electron density for the stalk domain was not observed, intramolecular tetrameric heads could not be distinguished from intermolecular packing interactions (Fig. S2A). In the crystals of the current PIV5 HN structure, the heads in the down position form crystal packing interactions with the heads in the up position of a neighboring HN tetramer using the previously observed DOD_1_ interface (Fig. S2 B-C). Therefore, neighboring HN molecules interact through the DOD_1_ interface in both of these crystal forms. Given the close spacing of HN molecules at the viral surface, it is possible that adjacent molecules could interact in a similar way serving to coordinate the function of interconnected tetramers. A similar observation was noted for MeV H through the formation of higher order structures on Blue Native PAGE gels. However, we note that such coordination is not required for syncytia formation given that headless PIV5 HN promotes cell-cell fusion at similar levels to wt .

**REFERENCES**

1. Yuan P, Thompson T, Wurzburg BA, Paterson RG, Lamb RA, et al. (2005) Structural studies of the parainfluenza virius 5 hemagglutinin-neuraminidase tetramer in complex with its receptor, sialyllactose. Structure 13: 1-13.

2. Brindley MA, Plemper RK (2010) Blue native PAGE and biomolecular complementation reveal a tetrameric or higher-order oligomer organization of the physiological measles virus attachment protein H. J Virol 84: 12174-12184.

3. Bose S, Zokarkar A, Welch BD, Leser GP, Jardetzky TS, et al. (2012) Fusion activation by a headless parainfluenza virus 5 hemagglutinin-neuraminidase stalk suggests a modular mechanism for triggering. Proc Nat Acad Sci USA 109: E2625-E2634.
